# For making a declaration of countermeasures against the falling birth rate from the Japanese Society for Hygiene: summary of discussion in the working group on academic research strategy against an aging society with low birth rate

**DOI:** 10.1186/s12199-019-0768-x

**Published:** 2019-03-05

**Authors:** Kyoko Nomura, Kanae Karita, Atsuko Araki, Emiko Nishioka, Go Muto, Miyuki Iwai-Shimada, Mariko Nishikitani, Mariko Inoue, Shinobu Tsurugano, Naomi Kitano, Mayumi Tsuji, Sachiko Iijima, Kayo Ueda, Michihiro Kamijima, Zentaro Yamagata, Kiyomi Sakata, Masayuki Iki, Hiroyuki Yanagisawa, Masashi Kato, Hidekuni Inadera, Yoshihiro Kokubo, Kazuhito Yokoyama, Akio Koizumi, Takemi Otsuki

**Affiliations:** 1 Department of Public Health, Akita University Graduate School of Medicine, Akita, Japan; 2 Department of Public Health, Kyorin University Gender Equality Promotion Office, Kyorin University School of Medicine, Tokyo, Japan; 3 Hokkaido University Center for Environmental and Health Sciences, Sapporo, Japan; 4 Department of Maternal Nursing, Division of Nursing, National Defense Medical College, Saitama, Japan; 5 Department of Hygiene, Kitasato University School of Medicine, Kanagawa, Japan; 6 Center for Health and Environmental Risk Research, National Institute for Environmental Studies, Ibaragi, Japan; 7 Institute of Decision Science for a Sustainable Society, Kyushu University, Fukuoka, Japan; 8 Graduate School of Public Health, Teikyo University, Tokyo, Japan; 9 Health Care Center, The University of Electro-Communications, Tokyo, Japan; 10 Research Center for Community Medicine and Department of Public Health, Wakayama Medical University School of Medicine, Wakayama, Japan; 11 Department of Environmental Health, University of Occupational and Environmental Health, Fukuoka, Japan; 12 Graduate School of Health Care and Nursing, Juntendo University, Chiba, Japan; 13 Graduate School of Global Environmental Studies, Kyoto University, Kyoto, Japan; 14 Department of Occupational and Environmental Health, Nagoya City University Graduate School of Medical Sciences, Nagoya, Japan; 15 Department of Health Sciences, School of Medicine, University of Yamanashi, Yamanashi, Japan; 16 Department of Hygiene and Preventive Medicine, Iwate medical University, Iwate, Japan; 17 Department of Public Health, Faculty of Medicine, Kindai University, Osaka, Japan; 18 Department of Public Health and Environmental Medicine, The Jikei University School of Medicine, Tokyo, Japan; 19 Department of Occupational and Environmental Health, Nagoya University Graduate School of Medicine, Nagoya, Japan; 20 Department of Public Health, Faculty of Medicine, University of Toyama, Toyama, Japan; 21 Department of Preventive Cardiology, National Cerebral and Cardiovascular Center, Osaka, Japan; 22 Department of Epidemiology and Environmental Health, Faculty of Medicine, Juntendo University, Tokyo, Japan; 23 Kyoto Hokenkai, Kyoto, Japan; 24 Department of Hygiene, Kawasaki Medical School, Okayama, Japan

**Keywords:** Child-maternal health, Environmental exposure, Japanese Society for Hygiene, Low birth rate Maternal nutrition, Reproductive health, Social work environment, Socioeconomic factors

## Abstract

In 1952, the Japanese Society for Hygiene had once passed a resolution at its 22nd symposium on population control, recommending the suppression of population growth based on the idea of cultivating a healthier population in the area of eugenics. Over half a century has now passed since this recommendation; Japan is witnessing an aging of the population (it is estimated that over 65-year-olds made up 27.7% of the population in 2017) and a decline in the birth rate (total fertility rate 1.43 births per woman in 2017) at a rate that is unparalleled in the world; Japan is faced with a “super-aging” society with low birth rate. In 2017, the Society passed a resolution to encourage all scientists to engage in academic researches to address the issue of the declining birth rate that Japan is currently facing. In this commentary, the Society hereby declares that the entire text of the 1952 proposal is revoked and the ideas relating to eugenics is rejected. Since the Society has set up a working group on the issue in 2016, there have been three symposiums, and working group committee members began publishing a series of articles in the Society’s Japanese language journal. This commentary primarily provides an overview of the findings from the published articles, which will form the scientific basis for the Society’s declaration. The areas we covered here included the following: (1) improving the social and work environment to balance between the personal and professional life; (2) proactive education on reproductive health; (3) children’s health begins with nutritional management in women of reproductive age; (4) workplace environment and occupational health; (5) workplace measures to counter the declining birth rate; (6) research into the effect of environmental chemicals on sexual maturity, reproductive function, and the children of next generation; and (7) comprehensive research into the relationship among contemporary society, parental stress, and healthy child-rearing. Based on the seven topics, we will set out a declaration to address Japan’s aging society with low birth rate.

## Introduction

According to the 50th anniversary of the Japanese Society for Hygiene, even before, the issue of an aging society with low birth rate had surfaced in Japan; the Society had already passed a resolution at its 22nd symposium on population control in 1952 to deliver the following proposal to the Ministry of Health and Welfare authorities:

“...the decline in mortality rates [...] is further accelerating the process of overpopulation in Japan. What this means for the future population is a decline in the amount of nutrition they will each receive, weaker physical health, and an increase in cases of tuberculosis and other diseases... Therefore, the most important thing that the government needs to do [...], in the aim of maintaining people’s heath from a public health stance, is to direct people to adjust the number of births [...] consciously. The Society [...] sincerely hopes that the government’s targets (for promoting the use of birth control) will be modified along the lines of cultivating a healthier population. This concludes our proposal.”

An additional resolution sets out that “in setting up (cooperating organizations to ensure the effective dissemination of birth control), the Society would like to be seen as a leading body because it has been interested in this issue for a number of years and has carried out considerable research.”

In other words, even after the new constitution, setting out fundamental human rights had been enacted; the Society was recommending birth control and the suppression of population growth based on the idea of cultivating a healthier population in the area of eugenics.

Over half a century has now passed since this recommendation; Japan is witnessing an aging of the population (it is estimated that over 65-year-olds made up 27.7% of the population in 2017) and a decline in the birth rate (total fertility rate 1.43 births per woman according to 2017 statistics) at a rate that is unparalleled in the world; Japan is faced with a “super-aging” society with low birth rate. Against such a backdrop, the Japanese Society for Hygiene passed a resolution at its 2017 annual general meeting to encourage all scientists to engage in academic researches to address the issue of the declining birth rate that Japan is currently facing.

It cannot be overlooked that the 1952 resolution (the proposal to the Ministry of Health and Welfare and related authorities concerning population health) was a proposal based on eugenics. The resolution set out the need for a perspective on eugenic protection, arguing that the Ministry of Health and Welfare is setting birth control targets that just emphasized the protection of maternal health which could lead to serious misunderstandings and also constitute a basis for committing the error of reverse selection. The Japanese Society for Hygiene does not advocate human rights violations based on eugenics, such as the forced sterilizations that occurred under the old Eugenic Protection Law (1948–1996). The Society, therefore, hereby declares that the entire text of the 1952 proposal is revoked and the ideas relating to eugenics is rejected.

Before making a declaration, the Society has set up a working group on strategies to address the declining birth rate, made up of members of the Society. Since the 2016 annual general meeting, there have been three symposiums and side events on the topic, and these events have enabled the Society to conduct discourse from a range of different angles. Working group committee members also began publishing a series of articles on the topic in the Japanese Journal of Society for Hygiene (i.e., Nihon Eiseigaku Zasshi) in January 2018. This paper primarily provides an overview of the findings from the symposiums and published articles, which will form the scientific basis for the Society’s declaration on the issue of Japan’s aging society with low birth rate.

## Improving the social and work environment to balance between the personal and professional life

In recent years in Japan, changes in the social and economic environment as well as in individual values, such as an increase in young people and on low incomes, have meant that a growing number of people either never marry or marry for the first time at a later age [1]. Further, even those who marry are not having the ideal number of children that they wish to have. Official statistics have shown that in addition to the economic cost of bringing up and educating children, the reasons for this include physical issues of infertility associated with women having children later, the burden of caring for a child, difficulties in combining childcare with a career, and differing values between the sexes [2]. Strategies to address the trend of having fewer children include creating social structures that enable women to conceive naturally at an appropriate age and give birth to healthy children, as well as to secure time for childcare without having to give up work. To this end, policies to support families need to move away from the male breadwinner model and toward the earner-career model, with more benefits in kind provided alongside cash wages [3, 4]. In addition, it is recommended that continued education is provided on the reproductive health for both men and women, and research carried out to assess the cost and effectiveness of medical care and policies surrounding childbirth [5, 6].

## Proactive education on reproductive health

It is a woman’s fundamental human right to choose and determine (the right to self-determination) whether to marry, whether to have children, when to have children, how many children to have, and how much of a gap to leave between each child. To consider and come to a decision on this, women need accurate information [7]. Fertility depends on age and begins to decline earlier in women than in men. The risk of miscarriage and obstetric complications in expectant and nursing mothers also increases with age [8]. Having accurate information gives women the opportunity to decide for themselves what they want to do with their lives [9–12]. It is important to provide information on the following matters: the existence of an appropriate age for a healthy and safe birth, the harmful effects of being underweight or smoking in women of childbearing age, the risk of infertility associated with sexually transmitted diseases, the high cost and low success rate of infertility treatment, the way to have good sexual relations, the precious experience of having children, and the pleasure of raising children. Thus, there is a need to create social support systems that would enable women who want to have children to do so.

## Children’s health begins with nutritional management in women of reproductive age

The Developmental Origins of Health and Disease (DOHaD) hypothesis, whereby a woman of reproductive age being underweight creates a state of starvation in the intrauterine environment and causes an epigenetic modification of the fetus, has become a topic of interest in recent years [13]. Underweight mothers are at risk of giving birth to infants with low birth weight as well as an increased risk of childhood obesity and lifestyle diseases such as diabetes later in life [14]. Further, women being underweight when they are young increases their risk of osteoporosis in the future, which can lead to fractures, being bedridden, or dementia [15, 16]. However, it is not only being underweight that is a problem; international and Japanese reports have also shown that anemia, being overweight, and obesity are associated with an increased risk of giving up breastfeeding at an early stage [17–19]. It is strongly recommended that health-related initiatives must ensure that all health care professionals involved in women’s health are acutely aware of the importance of nutritional management in women of reproductive age [20].

## Workplace environment and occupational health

On the issue of the health of women of childbearing age, and from the perspective of safeguarding fertility, it is essential to promote research in overwork and other areas of occupational health. Occupational health professionals should be trained particularly on mental health issues before and during pregnancy [21]. Further, working women undergoing infertility treatment are subject to a considerable burden regarding their physical and mental health, time, and finances [22]. Thus, further studies are recommended as there is still a considerable scope for investigating the kind of support that would be desirable. In terms of compatibility with treatment of diseases including cancer, it is essential that women can secure a certain amount of sick leaves and then supported to return to work at an early stage, as well as receive a follow-up support that also covers mental health issues after they have returned to work following a physical disorder such as cancer [23]. Particularly, as there is a higher incidence of cancer by age in women in the 30–40 years age group, there is a need for further research into the kind of support that is needed for women to cope with cancer alongside pregnancy, child-rearing, and working [24, 25]. Going forward, about establishing systems alongside the support provided for workers receiving medical treatment to prevent a condition from worsening, it is important to work in cooperation with occupational health professionals and members of the human resources and general affairs divisions at the workplace to promote staffing and other structural measures [26, 27]. Further research is recommended to demonstrate the scientific basis of such activities.

## Workplace measures to counter the declining birth rate

Workplace structures that provide a choice of working styles for women wanting to conceive or give birth and for couples with children also need to be extended to non-permanent staff and some small medium enterprises. It is recommended that measures are put in place to create a workplace environment that enables both parents to continue working. These measures include securing regular employment for men and women, replacing long working hours with efficient working systems, giving both men and women the option of childcare leave, shorter working hours or home teleworking, providing a nursery or using babysitters, and providing workplace support to enable couples to undergo infertility treatment alongside their work [28]. Many women may prefer for their colleagues not to know of any general medical treatment they may be receiving, especially infertility treatment. It is, therefore, necessary to give particular consideration to protecting personal information when setting up and operating employee leave systems. From an industrial health perspective, it is recommended that a workplace culture that accepts and supports pregnant women be created, that the Ministry of Health, Labour and Welfare’s maternity health management guidance contact cards are proactively used, and that restrooms are introduced to enable women to continue breastfeeding [29, 30].

## Research into the effect of environmental chemicals on sexual maturity, reproductive function, and the children of next generation

The third biggest reason women cite for not having their intended number of children is simply the inability to do so, representing 23.5% [31]. Scientific findings to date suggest that exposure to the increasing volume and variety of environmental chemicals could disrupt the next generation’s sex hormones in humans [32–34]. Workplace exposure to toxic substances could also have harmful effects on the reproductive function of workers of both sexes as well as on their children of the next generation [35]. Regular exposure to chemicals in daily life is also reported to have an effect on reproductive function. In other words, exposure to environmental chemicals with endocrine-disrupting properties might have harmful effects on the reproductive glands, reduce human fertility in both sexes, and be a cause of infertility in the future. It is important to carry out a range of studies from different perspectives and to evaluate the effects of simultaneous exposure to many substances including novel and alternative compounds that are developed and produced on a daily basis. Such studies should also examine public policy costs relating to eliminate or diminish emissions and restrictions on environmental chemicals.

## Comprehensive research into the relationship among contemporary society, parental stress, and healthy child-rearing

Women experience a huge burden by giving birth and becoming a mother, with plenty of mothers suffering from stress and anxiety. In particular, women who are pregnant or bringing up children tend to tire more easily, both physically and mentally, and are more prone to feeling isolated or stressed compared with before having children. Stress can be a causal factor in a wide range of illnesses. It is widely accepted that psychological stress affects the development and exacerbation of allergic disorders. In particular, parental psychological stress can also affect allergic reactions in infants who have a close relationship with their parents [36–39]. It is recommended that comprehensive academic and interdisciplinary studies that incorporate a range of specializations are carried out to investigate the effect of contemporary society and parental stress not only on allergic diseases but also on children’s growth and development [40] and that the findings are also communicated to the public.

## For making a declaration of countermeasures against the falling birth rate from the Japanese Society for Hygiene

**Fig. 1:**
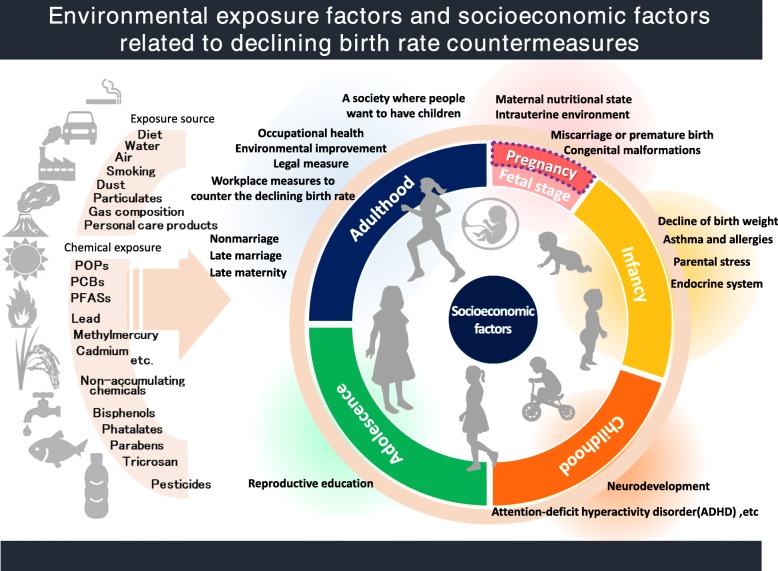
Miyuki Iwai-Shimada: trends in research relating to the link between and child health [41]

The time has come to give serious thought as to how best to protect the health of the children of the next generation amid the rapid decline in the working age population that is the backbone of Japan’s economic foundation (Fig. 1). Approximately 30% of the Japanese Society for Hygiene’s members are women, even in the field of natural sciences and medicine, with many studies resulting in hypotheses being developed from women’s or mothers’ perspectives and spanning a wide range of designs from experimental to epidemiological studies of groups. The Japanese Society for Hygiene believes it is essential to exploit the features of such a unique academic organization and keep the public informed in a way that is easy to understand regarding academic studies in fields such as environmental, reproductive, maternal and child, and occupational health.

## Abbreviations

PCBs: Polychlorinated biphenyls
PFASs: Poly- and perfluoroalkyl substances
POPs: Persistent organic pollutants
